# Effect of botanical powders and the assassin bug, *Alloeocranum biannulipes* Mont. and Sign. (Hemiptera: Reduviidae) against *Dinoderus porcellus* Lesne (Coleoptera: Bostrichidae) infesting yam chips

**DOI:** 10.12688/openresafrica.15173.2

**Published:** 2025-01-02

**Authors:** Yêyinou Laura Estelle LOKO, Joelle TOFFA, Innocent DJEGBE, Armand VODOUNNON, Antonio SINZOGAN, Kitherian SAHAYARAJ, Manuele TAMO

**Affiliations:** 1 Laboratoire de Zoologie Appliquée et Santé des Végétaux (ZASVE), Ecole Nationale Supérieure des Biosciences et Biotechnologies Appliquées (ENSBBA), Université Nationale des Sciences, Technologies, Ingénierie et Mathématiques (UNSTIM), Dassa-Zoumé, Collines, BP 14, Benin; 2 Ecole Normale Supérieure de Natitingou, Université Nationale des Sciences, Technologies, Ingénierie et Mathématiques (UNSTIM), BP 72 Natitingou, Benin; 3 Faculty of Agronomic Sciences, Universite d'Abomey-Calavi, Abomey-Calavi, 03 BP 2819, Benin; 4 Crop Protection Research Centre (CPRC), Department of Zoology, St. Xavier’s College (Autonomous), Palayamkottai, Tamil Nadu, 627002, India; 5 International Institute of Tropical Agriculture (IITA-Benin), Abomey-Calavi, 08 BP 0932, Benin

**Keywords:** Yam beetle, Dioscorea spp., Bioactivity, Farm storage, Integrated pest management

## Abstract

**Background:**

*Dinoderus porcellus* Lesne (Coleoptera: Bostrichidae) is the main pest of stored dried yam chips that causes significant losses in less than 3 months. The assassin bug, *Alloeocranum biannulipes* (Montrouzier & Signoret) (Hemiptera: Reduviidae) and the African mahogany ( *Khaya senegalensis* (Desv.) A. Juss. (Meliaceae)), the ackee ( *Blighia sapida* K. Koenig (Sapindaceae)), and bridelia ( *Bridelia ferruginea* Benth. (Euphorbiaceae)) leaf powders have proven to be efficient in the control of this pest.

**Methods:**

This study aims to evaluate the compatibility of the leaf powders of these medicinal plants and the predator *A. biannulipes* in the integrated management of *D. porcellus* under laboratory and farm conditions. Various leaf powders were tested at a concentration of 6% (w/w) with or without the predator. Infested yam chips without any treatment served as negative control and those mixed with a synthetic insecticide as positive control. The mortality rate of *D. porcellus* was recorded under laboratory conditions. While, the dynamic population of *D. porcellus*, their damage, and weight loss of yam chips were recorded 8 weeks after treatment under farm conditions.

**Results:**

The results revealed that no combination of leaf powders and predators could induce complete mortality of *D. porcellus* like the synthetic insecticide. No significant difference in terms of the survival of *A. biannulipes* exposed to botanical powders was observed compared to the positive control. Under farm conditions, *B. ferruginea* leaf powder showed a sub-lethal effect on the predator *A. biannulipes* and no impact on the abundance of *D. porcellus*. However, the survival of *D. porcellus* was significantly reduced by the combination of *K. senegalensis* leaf powder and *A. biannulipes,* which did not allow the reproduction of the predator.

**Conclusions:**

Our results suggest that an augmentative biological control program with the release of *A. biannulipes* after the introduction of *K. senegalensis* leaf powder is practicable for the management of *D. porcellus* in yam chips.

## Introduction

Yam ( *Dioscorea* spp.) is a staple food, and a source of medicine for West African populations ( Wumbei *et al.*, 2022). The Republic of Benin is the fourth-largest yam tuber-producing country with an estimated production in 2021 of 3,203,165.88 tons ( FAO, 2021). Cultivated for their fresh edible tubers, yam is instilled in the Beninese traditions and plays a key role both economically and socioculturally ( Baco *et al.*, 2007). Unfortunately, the high perishability of tubers makes yam a seasonal crop and leads to significant post-harvest losses of up to 85% of stocks ( Babajide *et al.*, 2008). To overcome these post-harvest losses, the women traditionally transform the tubers into yam chips after pre-cooking and drying in the sun ( Hounhouigan *et al.*, 2003). However, stored yam chips are severely damaged by the Bostrichid beetle, *Dinoderus porcellus* Lesne, which by feeding, reduces them to an inedible powder causing significant losses ( Loko *et al.*, 2022). The use of chemical insecticide is widely done by farmers to protect stored yam chips despite their adverse impact on the environment and human health ( Loko *et al.*, 2013). Therefore, developing eco-friendly control methods against *D. porcellus* is urgent for efficient yam chips protection.

Biological control using predators and medicinal plants with insecticidal effects is recognized as a less expensive and effective method of controlling insect pests, allowing not only the regulation of their populations but also reducing health and environmental risks ( Ratto *et al.*, 2022). The assassin bug, *Alloeocranum biannulipes* Mont. and Sign. (Hemiptera: Reduviidae) has proven to be a natural enemy that can be used in a biological control program against *D. porcellus* ( Loko *et al.*, 2017; Loko *et al.*, 2019). This predator has also proven to be an effective biological agent in the management of the main pest of cassava chips, the Bostrichid beetle, *Prostephanus truncatus* (Horn) ( Loko *et al.*, 2020). Furthermore, powders from the leaves of *Bridelia ferruginea* (Phyllanthaceae), *Blighia sapida* (Sapindaceae), and *Khaya senegalensis* (Meliaceae) used by farmers to control insects in stored yam chips are known to have insect repellent and insecticide properties against *D. porcellus* ( Loko *et al.*, 2017). The combination of the leaf powders of these three medicinal plants and varietal resistance has proven to be beneficial for the integrated management of the yam chips beetle ( Loko *et al.*, 2018). However, the compatibility of combinations between these three medicinal plants with the predatory *A. biannulipes* within an integrated management strategy of *D. porcellus* remains unknown. Integrated pest management combining biological control agents and botanical insecticides proves to be a promising alternative for the management of storage insects ( Schöller *et al.*, 1997; Weaver & Subramanyam, 2000). Combining biological control agent with botanical insecticides is more compatible than combining it with synthetic insecticides ( Noureldeen *et al.*, 2022; Sayed *et al.*, 2020). Therefore, the current study aims to evaluate the combined effect of leaf powders of *B. ferruginea*, *B. sapida*, and *K. senegalensis* and the predator *A. biannulipes* on the management of *D. porcellus*.

## Methods

### Mass-rearing of *D. porcellus*

The rearing of *D. porcellus* was carried out on yam chips of the Kokoro local varieties that were purchased at the Dantokpa market (longitude 2° 25' 60" E, and latitude 6° 22′ 32″ N) in Cotonou, Benin. The purchased dried yam chips were placed in an oven set at 70 °C for two hours to be sterilized before use. The *D. porcellus* adults provided by the Laboratory of Applied Zoology and Plant Health (ZASVE) of the Ecole Nationale Supérieure des Biosciences et Biotechnologies Appliquées (ENSBBA) at Dassa-Zoumé in Benin. Twenty pairs of *D. porcellus* were introduced into plastic boxes (195 length × 135 width × 55 depth mm) containing 500 g of dried yam chips and covered by a fine mesh muslin fabric held by an elastic band. Two weeks after incubation under laboratory conditions (26 ± 2°C, RH 75 ± 5%, 12L/12D), *D. porcellus* adults were removed from the rearing boxes and the offspring were used for experiments.

### Mass-rearing of *A. biannulipes*

The initial stock of the assassin bug ( *A. biannulipes*) adults was collected in Ouédéme village (longitude 1°40'60" E, and latitude 6°42'0" N) from farmers’ rice storage. Mass-rearing of this reduviid was done in the laboratory in plastic boxes (355 × 100 × 40 mm) containing five hundred grams of dried yam chips to which were added 100 adults of *D. porcellus* of indeterminate sex. After one week, 20 adults of *A. biannulipes* of indeterminate sex were added to the rearing boxes containing the infested yam chips. The predators were removed from the plastic boxes two weeks after their release, and the offspring were used for further experiments.

### Preparation of botanical powders

Three medicinal plants including *B. ferruginea*, *B. sapida*, and *K. senegalensis* were collected from Abomey-Calavi district (latitude 6°26'0" N, and longitude 2°18'0" E). The identification of these plant species was done by the experts of the National Herbarium of Benin (University of Abomey-Calavi). The leaves sampled were carefully washed and shade-dried at room temperature (to avoid the inactivation of bioactive compounds by the sunlight) until they were crisp. A laboratory blender was used to transform dried leaves into powders. The obtained powder was sieved through a fine-mesh sieve (300 µm). The fine powders obtained were packaged in hermetically sealed black boxes and covered with aluminium foil to prevent photodegradation. The boxes were maintained under ambient laboratory conditions (26 ± 2°C, RH 75 ± 5%, 12L/12D), until use.

### Bioassays under laboratory conditions

The interaction between the predator *A. biannulipes* and botanical powders in the biological control of *D. porcellus* was assessed according to the methodology of Flinn *et al.* (2006) with slight modifications. Five pairs (5 male and 5 female) of *D. porcellus* adults (3 to 7 days old) were introduced into plastic experimental boxes (120 length × 85 width × 65 depth mm) containing 100 g of sterilized yam chips. The sex determination of *D. porcellus* adults was based on the morphology of the genitalia ( Aziz & Sitaraman, 1948). After 20 days, which correspond to the maximum time required for optimal larval development ( Oni & Omoniyi, 2012) or the first emergence of *D. porcellus* offspring ( John *et al.*, 2021), a couple of adult *A. biannulipes* (1–3 days old) and the leaf powders of the different plants at a rate of 6% (w/w) was added to the experimental boxes. The choice of this application rate was based on a previous study that showed at 6% (w/w) the leaf powders of all the tested plants cause a high mortality of *D. porcellus* adults ( Loko *et al.*, 2018). Yam chips infested with *D. porcellus* without predators served as a negative control. The positive control consisted of adding to experimental boxes a couple of *A. biannulipes* (1–3 days old), the synthetic insecticide Antouka Super® (SYNGENTA, United. Kingdom) (Permethrin 3g/kg + pyrimiphos 16g/kg; DP) at the recommended dose of 0.05% w/w, which has shown its effectiveness in protecting yam chips against *D. porcellus* in previous studies ( Loko *et al.*, 2017). Each box was closed with a muslin fabric to prevent unwanted insect movement. Experimental boxes were incubated under laboratory conditions (26 ± 2°C, RH 75 ± 5%, 12L/12D). The experimental design used was a completely randomized block with 4 repetitions. *Dinoderus porcellus* adult mortality data were taken at 1, 3, 5, 7, 14, and 21 days after treatment ( Othira *et al.*, 2009). The calculated mortality percentage was corrected with Schneider-Orelli's formula ( Püntener, 1981).

$$\mathrm{Corrected}\;\mathrm{mortality}=\frac{\left(\%\;\mathrm{of}\;\mathrm{death}\;\mathrm{in}\;\mathrm{treated}-\%\;\mathrm{death}\;\mathrm{in}\;\mathrm{control}\right)}{\left(100-\%\;\mathrm{death}\;\mathrm{in}\;\mathrm{control}\right)}\times 100$$

### Bioassays under farm storage with variable environmental conditions

The interaction between the botanical powders and the predator *A. biannulipes* for the management of *D. porcellus* in the stored yam chips was evaluated according to farmers’ storage practices ( Loko *et al.*, 2013). For the experiment, one kilogram of yam chips (9.7 ± 1.1 pieces of yam chips, each weighing 93.4 ± 1.8 g on average) obtained at the Dantokpa market and previously sterilized were put in woven polypropylene bags (65 length × 48 width cm). Twenty adults (1–3 days old) of *D. porcellus* were added to each experimental woven bag. For each plant species, four treatments repeated 4 times were tested: infested yam chips (control), infested yam with leaf powder (6% w/w), infested yam chips with the predator, infested yam chips with the predator and the leaf powder (6% w/w). Twenty days after the introduction of *D. porcellus* adults, two couples of *A. biannulipes* (1–3 days old) were added to treatments with predators. Sex determination was made based on differences in genitalia between male and female adults of *A. biannulipes* ( Loko *et al.*, 2019). The bags were sealed with heavy string and arranged in completely randomized experimental blocks in a farmer’s room at the Benin station of the International Institute of Tropical Agriculture located at Abomey-Calavi. This south region is characterized by an equatorial climate with alternating two dry seasons and rainy seasons. Precipitation varies between 900 and 1200 mm with an average of 1100 mm. During the experiments, the temperature varied from 25 to 29°C with an average relative humidity of about 75 ± 5%. After eight weeks, the numbers of dead and live insects (larvae, nymphs, and adults) were noted. The damage was assessed using the visual yam chips assessment method described by Compton *et al.* (1993). This is a method of visually assessing damage based on the number of holes made by the insect pest when it feeds on yam chips. The yam chips were sieved to remove the flour, and weighed for the determination of lost weight compared to the initial weight.

### Data analysis

The data were transformed to guarantee the homogeneity of the variances before being used for an GLM Repeated Measure analysis of variance (ANOVA) using SPSS software, version 23.0. Significant differences between means were determined by the Student-Newman-Keuls test at 5% probability. Kaplan–Meier analysis was used to estimate the mean survival time and survival curves of *A. biannulipes* in the different treatments using the MedCalc software version 17. The log-rank test was used to determine the statistical significance of the differences in the longevity of *A. biannulipes* adults.

## Results

### Combined effect of the botanical powders and the predator on *D. porcellus* i(laboratory conditions)

Table 1 presents the percentage corrected mortalities of *D. porcellus* adults after application of the various leaf powders of *K. senegalensis*, *B. sapida*, and *B. ferruginea* with or without the predator *A. biannulipes*. On day 1 after treatment, the mortality rate of *D. porcellus* was not significantly (df = 27; F = 0.74; p > 0.05) different between treatments. On the other hand, on the third day a significant difference (df = 27; F = 3.92; p < 0.001) was observed between the treatments with the synthetic insecticide, which showed the highest mortality (77.20 ± 16.10) observed compared to the other treatments ( Table 1). In addition, the mortality rate of *D. porcellus* differed significantly (df = 1; F = 6.12 p < 0.05) between the treatments with and without predators. However, no significant (df= 2; F = 0.82; p > 0.05) interaction between the different combinations of medicinal plants and the predator *A. biannulipes* was observed in terms of *D. porcellus* mortality. The same trend was observed on the fifth day with a very highly significant difference (df = 27; F = 12.67; p < 0.0001) observed between the treatments. The synthetic insecticide presented the highest mortality (93.80 ± 12.50) while the combination of *B. ferruginea* and *A. biannulipes* presented the lowest mortality (46.90 ± 8.60). No significant interaction (df = 2; F = 1.94; p > 0.05) was observed between the different combinations of botanical powders and *A. biannulipes*. It was only on the seventh day that a significant interaction (df = 2; F = 9.39; p < 0.001) between the leaf powders of the various medicinal plants and the predator *A. biannulipes* was observed about *D. porcellus* mortality. This interaction between plants and predators persisted significantly at 14 days (df = 2; F = 11.94; p < 0.0001) and day 21 (df = 2; F = 15.08; p < 0.0001). Therefore, a highly significant difference in *D. porcellus* mortality was observed between the different treatments on the seventh (df = 27; F = 18.25; p < 0.0001), fourteenth (df = 27; F = 12.67; p < 0.0001), and twenty-first (df = 27; F = 14.36; p < 0.0001) days after treatment. The synthetic insecticide showed 100% mortality of *D. porcellus* by the seventh day, and there was a positive interaction between *K. senegalensis* leaf powder and *A. biannulipes* compared with treatment with botanical powder alone. However, the interaction between *B. ferruginea* leaf powder and the predator *A. biannulipes* was negative from the very first days after treatment.

**Table 1.** Mean corrected mortality (± standard deviation) of *D. porcellus* after treatment with botanical powders in the presence or absence of the assassin bug *A. biannulipes* at an interval of 1 to 21 days after treatments (DAT) under laboratory conditions.

Treatments	Corrected mortality rate (%) after different days of observations					
	1	3	5	7	14	21
**Synthetic insecticide**	33.23 ± 11.17a	77.23 ± 16.06b	93.75 ± 12.50c	100.00 ±0.00c	100 ± 0.00b	100.00 ± 0.00b
***B. sapida* + predator**	24.05 ± 35.21a	56.70 ± 5.13ab	59.82 ± 3.09b	61.67 ±3.33b	66.25 ± 13.77b	68.75 ± 12.50b
***B. sapida***	20.14 ± 28.85a	62.95 ± 14.39ab	72.77 ±12.49b	70.83 ± 12.58bc	71.25 ± 14.36b	75.00 ± 0.00b
***B. ferruginea* + predator**	20.93 ± 24.95a	43.30 ± 5.13a	46.87 ± 8.55a	23.33 ± 17.64a	21.25 ± 16.52a	25.00 ± 20.41a
***B. ferruginea***	31.20 ± 13.01a	59.82 ± 3.09ab	63.39 ± 5.92b	61.67 ± 3.33b	77.50 ± 20.61b	90.75 ± 11.40b
***K. senegalensis* +** **predator**	37.90 ± 16.04a	59.82 ± 15.80ab	70.09 ± 5.33b	90.00 ± 11.55bc	87.50 ± 14.43b	93.75 ± 12.50b
***K. senegalensis***	47.82 ± 6.16a	66.96 ± 5.15b	73.21 ± 2.06b	85.83 ± 9.57bc	82.50 ± 11.90b	87.50 ± 14.43b

In a column, the means followed by different letters are significantly different at the 5% level.

**Figure 1.**
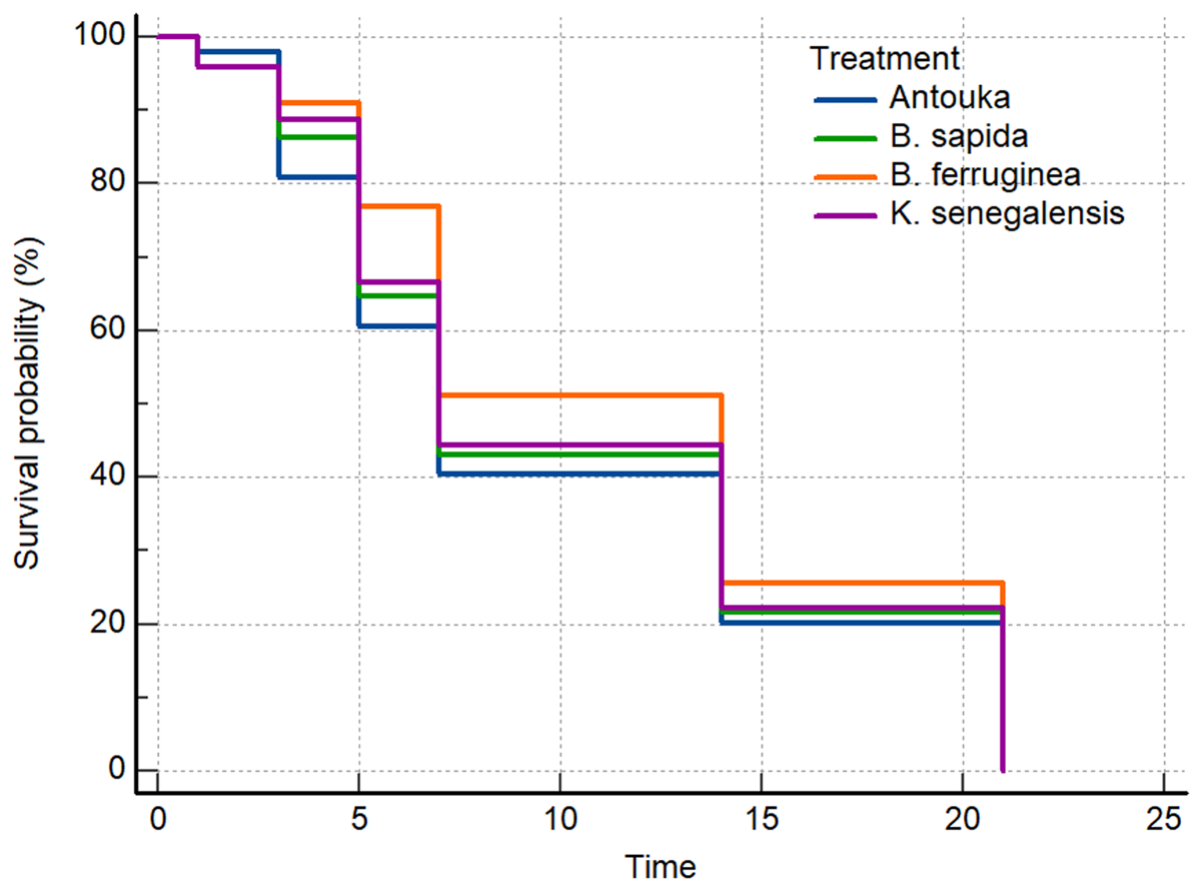
Survival curves of *A. biannulipes* adults exposed to yam chips treated with botanical powder, and the synthetic insecticide under laboratory conditions (time in days).

The restricted mean survival time of the predator *A. biannulipes* adults after exposure to treated yam chips with the different botanical powders showed no significant difference in the function of plant species. Indeed, the restricted mean survival time of *A. biannulipes* adults varied from 10.00 ± 1.10 days (synthetic insecticide) to 11.70 ± 1.10 days ( *B. ferruginea*). The comparison of survival curves (Logrank test) showed no significant (chi-squared = 1,273, df = 3, p > 0.05) difference between treatments ( Figure 1).

### Combined effect of botanical powders and the predator on *D. porcellus* population (farm conditions)

After 8 weeks' storage of yam chips, *D. porcellus* at various stages of development was found in the different treatments ( Table 2). The population of *D. porcellus* has fluctuated significantly depending on the different treatments. Indeed, significantly (df = 27; F=16.013; P= 0.000) fewer live *D. porcellus* larvae were found in yam chips treated with *K. senegalensis* leaf powder with or without predator. A highly significant difference (df = 27; F = 8.86; p < 0.0001) was observed between the different treatments and the control, which presented the highest average number of live *D. porcellus* nymphs (2.25 ± 0.63). The mean number of *D. porcellus* adults was significantly higher (df = 27; F = 11.68; p < 0.0001) in the control and in the treatment with *B. ferruginea* leaf powder with or without predators compared with the other treatments ( Table 2).

**Table 2.** Mean number of living *D. porcellus* and *A. biannulipes* (± standard deviation) present in yam chips treated with botanical powders in the presence or absence of the predator, 8 weeks after treatment under storage conditions.

Treatments	*D. porcellus*			*A. biannulipes*	
	Larvae	Nymphs	Adults	Adults	Nymphs
Control*	12.00 ± 1.08 c	2.25 ± 0.63 b	15.25 ± 0.85 d	-	-
**Predator + *B. sapida***	2.75 ± 0.63 b	0.00 ± 0.00 a	6.00 ± 0.58 ab	0.50 ± 0.57 a	0.50 ± 0.57 a
***B. sapida***	2.00 ± 0.41 b	0.00 ± 0.00 a	4.00 ± 0.91 a	-	-
**Predator + *B. ferruginea***	3.25 ± 0.25 b	0.75 ± 0.48 a	8.50 ± 1.85 bc	2.5 ± 0.57 b	2.00 ± 0.81 b
***B. ferruginea***	4.25 ± 0.48 b	1.00 ± 0.41 a	10.75 ± 1.49 cd	-	-
**Predator + *K. senegalensis***	0.25 ± 0.25 a	0.00 ± 0.00 a	3.75 ± 0.48 a	0.00 ± 0.00 a	0.00 ± 0.00 a
***K. senegalensis***	1.00 ± 0.58 a	0.00 ± 0.00 a	4.75 ± 0.63 ab	-	-

control: infested yam chips without any treatment. In a column, the means followed by different letters are significantly different at the 5% level.

Live adults and nymphs of the reduviid *A. biannulipes* were found in yam chips treated with *B. sapida* and *B. ferruginea* leaf powders combined with the predator after eight weeks of storage ( Table 2). Only yam chips treated with *K. senegalensis* leaf powder did not allow the reproduction of *A. biannulipes*. Yam chips treated with *B. ferruginea* leaf powder combined with adult predators showed significantly more nymphs (df = 11; F = 14.44; p < 0.001) and adults (df = 11; F = 22.59; p < 0.0001) of *A. biannulipes* after eight weeks of storage compared to the other treatments.

**Figure 2.**
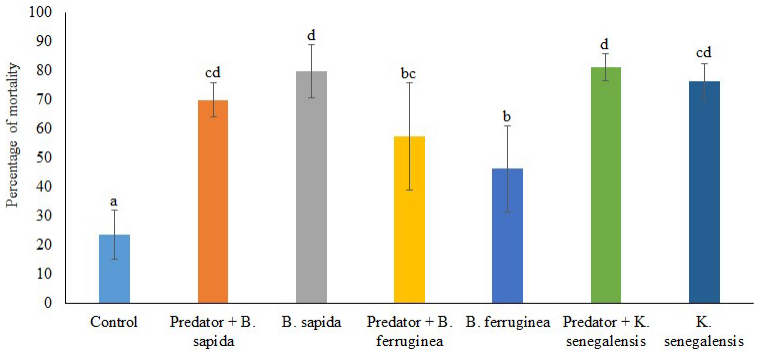
Percentage of *D. porcellus* mortality after treatment with botanical powders with and without the assassin bug *A. biannulipes*.

Figure 2 illustrates the mortality rate of *D. porcellus* induced by the botanical powders combined or not with the predator *A. biannulipes* in farm conditions. There is a very highly significant difference (df = 27; F=15.00; p < 0.0001) between the control and the other treatments in terms of *D. porcellus* mortality. The botanical powders used alone caused a high mortality of *D. porcellus* ranging from 46.25 ± 7.46% ( *B. ferruginea*) to 80.00 ± 9.12% ( *B. sapida*). Only the combinations of *K. senegalensis* with predator, as well as *B. ferruginea* with predator gave slightly higher mortality rates than the botanical powders used alone. However, the interaction between the different botanical powders and *A. biannulipes* concerning the *D. porcellus* mortality was not significant (df = 2; F = 2.21; p > 0.05).

**Figure 3.**
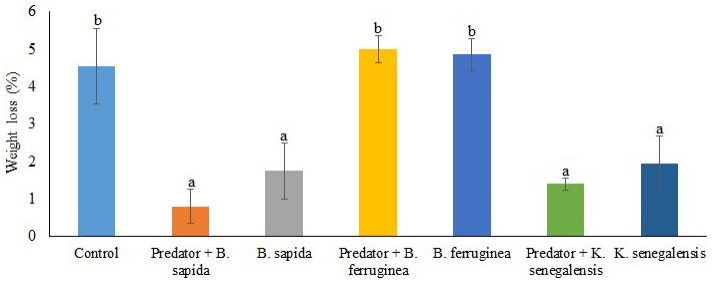
Weight loss induced by *D. porcellus* in yam chips mixed with botanical powders with and without the assassin bug *A. biannulipes*.

The results showed that the significantly (df = 27; F = 8.144; p < 0.0001) highest weight losses were recorded in the control treatment (4.55 ± 1.00%), chips treated with *B. ferruginea* powder alone (4.85 ± 0.43 %) or with a predator (5.00 ± 0.35%). On the other hand, the chips treated with *B. sapida* and *K. senegalensis* leaf powders in the presence or absence of the predator showed significantly (df = 27; F = 8.144; p < 0.0001) the lowest percentages of yam chips weight loss ( Figure 3). The plant species significantly (df = 3; F = 14.651; p < 0.0001) influenced the weight loss resulting from *D. porcellus* feeding. However, the interaction between the botanical powders with the predator *A. biannulipes* was not significant (df = 2; F = 1.03; p > 0.05) concerning the weight loss of yam chips.

**Figure 4.**
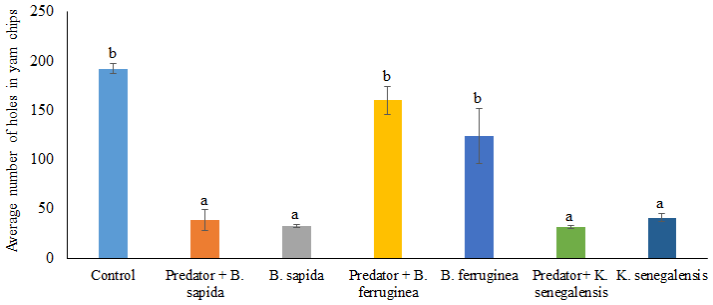
Damage induced by *D. porcellus* after treatment of yam chips with botanical powders in the presence or absence of the assassin bug *A. biannulipes*.

Figure 4 presents the damage (mean number of holes) induced by *D. porcellus* on yam chips when treated with the three botanical powders in the absence and presence of *A. biannulipes*. The average number of holes forged by *D. porcellus* in the yam chips varied significantly (df = 3; F = 26.21; p < 0.0001) depending on the plant species used for the treatment. Indeed, the average number of holes counted in the yam chips mixed with *B. ferruginea* leaf powder, used alone (123.75 ± 28.28) or with the predator (160.00 ± 14.31) was not significantly different from the control (192.25 ± 4.70). On the other hand, the average number of holes recorded in the yam chips treated with *B. sapida* and *K. senegalensis* leaf powders differed significantly from the control (df = 27; F = 15.01: p < 0.0001). Similar results were observed with the combination of *B. sapida* and *K. senegalensis* leaf powders with *A. biannulipes* ( Figure 4). However, there was no significant interaction (df = 2; F = 1.09; p > 0.05) between the leaf powders of the three medicinal plants and the predator *A. biannulipes* about the reduction of damage (number of holes) induced by *D. porcellus* to yam chips.

## Discussion

Under laboratory conditions, all treatments caused significant mortality of *D. porcellus* compared with the negative control, and this mortality increased with storage time. This is not surprising because it is known that all the tested plants have insecticidal properties against *D. porcellus* ( Loko *et al.*, 2017), and the effectiveness of the predator *A. biannulipes* as a biological control agent has been demonstrated ( Loko *et al.*, 2019). However, compared to the synthetic chemical insecticide, no treatment was able to induce total mortality of *D. porcellus*. Nevertheless, 7 days after treatment, no significant difference was observed between the mortality induced by this synthetic chemical insecticide and that of the leaf powder of *B. sapida*, as well as that of the combination of leaf powder of *K. senegalensis* and the predator. Toffa *et al.* (2021) showed that the synthetic insecticide, is more effective against *D. porcellus* by ingestion than by contact. In addition, this insecticide showed a very low repellence against *D. porcellus* ( Loko *et al.*, 2017; Toffa *et al.*, 2021). According to the literature, the active components of the used synthetic insecticide could act by affecting the insect nervous system ( Williamson *et al.*, 2023). However, to reduce the development of resistance to insecticides with active ingredient mixtures similar to the synthetic chemical used, Bawa *et al.* (2022) suggested integrating biological management methods.

After three days, the mortality of *D. porcellus* differed significantly between the treatments with predators and those without predators. This could suggest a synergy of action between botanical powders and predators in the control of *D. porcellus*. However, it was only after seven days that a significant interaction between the botanical powders and the predator was recorded. This could be explained by the strong repellent effect of these botanical powders ( Loko *et al.*, 2017), which deteriorated over the days and then allowed the action of the predator after post-application persistence. Lima *et al.* (2020) recorded the repellent action of botanical insecticides on the predatory stinkbug *Podisus nigrispinus* Dallas (Hemiptera: Pentatomidae). Smith and Perfetti (2020) studied the persistence of some botanical insecticides and revealed a minimum persistence time of 101 h. Consequently, it is important that further studies identify the volatile substances responsible for the repellent effect of these botanical powders, and explore the persistence time of their bioactivity to better define an integrated control method for *D. porcellus*.

Our results revealed that the bioactivity of botanical powders and the synthetic insecticide on the survival of *A. biannulipes* was similar under laboratory conditions. Indeed, these botanical powders have shown a lethal effect on *A. biannulipes* and act mainly by obstructing the stigmata of insects causing their death by suffocation. In addition, one or more active compounds present in the leaf powders of these plants could have a lethal effect on *A. biannulipes*. Botanical insecticides have been shown to deter the olfaction of assassin bugs such as *Rhynocoris fuscipes*, and *Rhynocoris marginatus* adults ( Jasmine *et al.*, 2012). Sahayaraj and Paulraj (1999) also observed the toxicity of plant-based pesticides through the contact and ingestion of the predator *R. marginatus*. Therefore, it is important to determine the mechanism of lethal action of the tested botanical powders.

Under farm conditions, there is a low insecticidal effect of the *B. ferruginae* leaf powder on *A. biannulipes*, and their interaction did not effect on the development of *D. porcellus*, which was able to cause similar damage to the control test. This result suggests that *B. ferruginea* powder had a sub-lethal effect on the predator *A. biannulipes,* preventing it from controlling the *D. porcellus* populations. In fact, it has been shown that botanical insecticides can modify the ethology of a natural enemy without killing it ( de Franca *et al.*, 2017; Jasmine *et al.*, 2012). It has been shown that the orientation of an insect predator could be disrupted by the sublethal concentration of the botanical insecticide ( Dai *et al.*, 2019). Furthermore, the insignificant insecticidal action of *B. ferruginae* leaf powder against *D. porcellus* could be explained by the rapid degradation of active components in the leaf powder under the effect of climatic conditions in the storage structure. As part of the development of an effective integrated control method for *D. porcellus*, it is important to identify and characterize the sublethal effects of the leaf powders of three medicinal plants on *A. biannulipes*.

A synergistic effect between *K. senegalensis* leaf powder and *A. biannulipes* in the management of *D. porcellus* was observed under laboratory and farm conditions. The mechanisms underlying this synergistic interaction are unclear, but there is evidence that *K. senegalensis* can influence the feeding and movements of *D. porcellus* ( Loko *et al.*, 2017), making it more susceptible to attack by *A. biannulipes*. Some studies have also proven the effectiveness of *K. senegalensis* leaf powders in controlling storage insect pests such as *Tribolium casteneum* Herbst (Coleoptera: Tenebrionidae) ( Bamaiyi & Bolanta, 2006), and *Sitophilus zeamais* L. (Coleoptera: Curculionidae) ( Oyewale *et al.*, 2014). However, *K. senegalensis* leaf powders had both lethal and sub-lethal effects on *A. biannulipes* by preventing its reproduction. Indeed, the sublethal dose of bioinsecticides could interfere with the chemical communication system of the biological agent and reduce its chance of reproduction ( de Franca *et al.*, 2017). Some studies have demonstrated the egg-laying deterrent properties of *K. senegalensis* products on the Bruchid *Callosobruchus maculatus* Fabricius ( Babarinde & Ewete, 2008; Bamaiyi *et al.*, 2006), and *Sitophilus oryzae* L. (Coleoptera: Curculionidae) ( Obeng-Ofori & Akuamoah, 2000). Our results, therefore, suggest that the use of *K. senegalensis* leaf powder combined with *A. biannulipes* in the control of *D. porcellus* should be done as part of an augmentative biological control program with the release of predators after application of the leaf powder.

*B. sapida* leaf powder, used alone and/or with the predator, caused significant mortality of *D. porcellus* in the laboratory and significantly reduced losses and damage under farm conditions. For long-term storage of yam chips, our results suggest the use of a combination of *B. sapida* and *A. biannulipes* because they presented a reduced population of *D. porcellus* after eight weeks of storage and offspring of *A. biannulipes*. However, we recommend the use of *B. sapida* leaf powder without a predator or a combination of *K. senegalensis* leaf powder and *A. biannulipes* for the short-term storage of yam chips.

It is known that plants with insecticidal effects can influence the sensory qualities of treated foods through the absorption of certain botanical compounds ( Guru-Pirasanna-Pandi *et al.*, 2018). The infestation of yam chips by insect pests ( Babarinde *et al.*, 2013) and the inclusion of indigenous additives ( Tanimola *et al.*, 2022) affect the quality and acceptability of the dough obtained from yam flour locally, known as 'Amala'. Therefore, it is important to evaluate the impact of plant powder residues on the sensory qualities of treated yam chips.

## Conclusions

In the framework of the development of an integrated control method against the main pest of stored yam chips, the combined effect of the predator *A. biannulipes* and the leaf powder of three medicinal plants was studied for the first time. Among the three tested combinations, *B. ferruginae* and *A. biannulipes* were not promising and, therefore, cannot be recommended for an integrated pest management program (IPM). Our results suggest that an augmentative biological control program with the release of *A. biannulipes* after the introduction of K. *senegalensis* leaf powder is practicable for the management of *D. porcellus* in yam chips. Further research is needed to examine the impact of plant powder residues on the sensory qualities of treated yam chips. 

## Declarations

### Ethics approval and consent to participate

Not applicable.

### Consent for publication

Not applicable.

## Data Availability

The data that support the findings of this study are openly available in “figshare” at https://doi.org/10.6084/m9.figshare.24083955.v1 Loko, Yêyinou Laura Estelle (2023). Database biocontrol botanical powders and predator. figshare. Dataset. https://doi.org/10.6084/m9.figshare.24083955.v1 This manuscript contains the laboratory and farm experiment on the integrated control *of D. porcellus* dataset.xlsx Data are available under the terms of the Creative Commons Attribution 4.0 International license (CC-BY 4.0).
