# Supplementary material for: Effect of botanical powders and the assassin bug, Alloeocranum biannulipes Mont. and Sign. (Hemiptera: Reduviidae) against Dinoderus porcellus Lesne (Coleoptera: Bostrichidae) infesting yam chips
Source: Open Res Afr. 2025 Jan 2;7:7. Originally published 2024 Jul 8. [Version 2] doi: 10.12688/openresafrica.15173.2 (PMC11775442; doi:10.12688/openresafrica.15173.2)
Supplement: Peer review history

# Reviewer report: Reviewer response for version 2

**DOI:** 10.21956/openresafrica.16855.r32067

**Published:** 2025-01-28

**Authors:** Sylvia Opiyo

**Affiliations:** 1 Murang'a University of Technology, Murang'a, Kenya

The work is clearly and accurately presented in well-defined sections as follows: abstract, keywords, background, objective methodology, results and discussion, and conclusion. The results are well presented in tables and figures (with statistical analysis). However, there is a need to include references in the methodology sections involving the mass-rearing of the test insects, especially if the methods were borrowed from someone's work.

Is the work clearly and accurately presented and does it cite the current literature?

Yes

If applicable, is the statistical analysis and its interpretation appropriate?

Yes

Are all the source data underlying the results available to ensure full reproducibility?

Yes

Is the study design appropriate and is the work technically sound?

Yes

Are the conclusions drawn adequately supported by the results?

Yes

Are sufficient details of methods and analysis provided to allow replication by others?

Yes

Reviewer Expertise:

Natural products chemistry;   insect pest control in stored crop/ food using bioactive  plant extracts

I confirm that I have read this submission and believe that I have an appropriate level of expertise to confirm that it is of an acceptable scientific standard.

# Reviewer report: Reviewer response for version 2

**DOI:** 10.21956/openresafrica.16855.r32028

**Published:** 2025-01-07

**Authors:** Guru P. N

**Affiliations:** 1 Department of Agricultural Entomology, Indian Council of Agricultural Research (ICAR)-Central Institute of Post-Harvest Engineering and Technology, Ludhiana, Punjab, India

Authors have addressed the suitable points with appropriate answers in the manuscript.

Is the work clearly and accurately presented and does it cite the current literature?

Partly

If applicable, is the statistical analysis and its interpretation appropriate?

Partly

Are all the source data underlying the results available to ensure full reproducibility?

Partly

Is the study design appropriate and is the work technically sound?

Yes

Are the conclusions drawn adequately supported by the results?

Partly

Are sufficient details of methods and analysis provided to allow replication by others?

Partly

Reviewer Expertise:

Agricultural Entomology

I confirm that I have read this submission and believe that I have an appropriate level of expertise to confirm that it is of an acceptable scientific standard.

# Reviewer report: Reviewer response for version 1

**DOI:** 10.21956/openresafrica.16338.r31526

**Published:** 2024-09-14

**Authors:** Samuel Adelani Babarinde

**Affiliations:** 1 Department of Crop and Environmental Protection, Ladoke Akintola University of Technology, Ogbomosho, Oyo, Nigeria

**Background**

*Dinoderus porcellus* Lesne (Coleoptera: Bostrichidae) is a major pest of stored yam chips. The article reports integrated pest management of the pest using its biological control agent (the assassin bug, *Aloecranum biannulipes *Mont. and Sign.) (Hemiptera: Reduviidae) and three botanical powders ( *Khaya senegalensis* (Desv.) A. Juss.), *Blighia sapida* K. Koenig and *Bridelia ferruginea* Benth. Two bioassays were carried out in the laboratory and under the farm conditions. The authors designed the experiments well and collected relevant data to establish logical conclusions.

 However, there are some fundamental issues which must be addressed to improve the quality of the article. I have included comments for the different segments of the manuscript for authors’ consideration.

**Abstract**

*Methods*

 1. Change ‘leaf powder of these ….’ to ‘leaf powders of three medicinal plants’

 2. Your methodology includes two bioassays (laboratory and field conditions). Incidentally, what you have in the Abstract does not succinctly capture that. Specifically, I suggest that you should make some clarifications: “Yam chips mixed with leaf powder from each species served as negative control” What would yam chips without any treatment be?

*Results*

 The results section also fails to briefly differentiate between the laboratory and field experiments.

*Conclusion*

 Consider the last sentence of the Conclusion and recast to make it more comprehensive.

**Introduction**

*The last three sentences of the Introduction*

 1.Remove ‘while’ at the beginning of the sentence.

 2. Recast the sentence beginning with ‘Indeed, compared…’ you may wish to consider the following: Combining biological control agent with botanical insecticides is more compatible than combining it with synthetic insecticides.

**Methods**

*Preparation of botanical powder*

 To what moisture content did you dry the botanicals or ‘just until crisp’? That information will support reproducibility of the research.

*Bioassay under laboratory conditions*

*1. *insert the word ‘to’. Let it read: … was assessed according **to **the …

 2. Let ‘plastic boxes’ be changed to … ‘plastic experimental boxes…’ and expunge ‘experimental boxes’ in parenthesis at the end of the sentence.

 3. Do not begin a sentence with an abbreviation. *D. porcellus *should be written in full at the beginning of a sentence to read *‘Dinoderus porcellus’.*

*Bioassays under farm storage with variable environmental conditions*

1. What of the negative control with plant powders which you mentioned in the Abstract?
2. You used woven polypropylene bags for the field experiment and you claimed that the bags were hermetically sealed. How feasible is it to achieve a hermetic condition with a woven bag? Or did you put an airtight inner layer in the woven bag?
3. Did you consider the immatures in the chips?

**Data analysis**

 In the last sentence under Data analysis, ‘The original data obtained were presented in the tables and figures’: Which data are you referring to? Is this sentence really necessary?

**Results**

1. Tidy up some typographical errors, omission of words; and reduce verbosity.

1. For instance, you may wish to consider the first sentence to be recast as follows: ‘Table 1 presents percentage corrected mortalities of *D. porcellus* ….’
2. Use ‘significantly (df = 27, …..) **different’. **Do not omit the word ‘different’

1. Which control are you refereeing to in Table 2? Indicate it as a footnote since you have different controls for your bioassays.
2. The df should be 27. You typed 2 somewhere on page 4 of 12 towards the end of the second column.
3. Where p=0.000, I suggest that you present it as p<0.0001.
4. Be consistent with the number of decimal places. For instance, 80±9.12% should be rendered as 80.00±9.12%.
5. Does *D. porcellus* have both larvae and nymphs? Is it a holometabolous or hemimetabolous insect?
6. Delete the period/point after the table title or figure legend, if such is not a sentence.

**Discussio**n

1. First paragraph 6 ^th^ line from the end of the paragraph: synthetic insecticide could be act by ..., Delete ‘be’
2. Third paragraph: Use ‘spiracles’ instead of ‘stigmata’. Stigmata is more appropriate for acarina than insects.
3. Third paragraph: The last sentence is too clumsy. Attempt to recast it. You may wish to consider this suggestion: Therefore, it is important to determine the mechanism of lethal action of the tested botanical powders.
4. Paragraph four: Recast: … interaction did not effect on the development … Recast.
5. **Some studies** have demonstrated the egg-laying deterrent properties of *K. senegalensis *products on the **Bostrichid**, *Callosobruchus maculatus* Fabricius (Bamaiyi et al. 2006)

1. *Callosobruchus maculatus *is not a Bostrichid, but a Bruchid.
2. For ‘some studies’, you need more than just one reference. You may wish to add Babarinde and Ewete (2008) to Bamaiyie et al., 2006; with the following citation: Babarinde, S. A., & Ewete, F. K. (2008). Comparative bioactivity of three Khaya species (Meliaceae) against Callosobruchus maculatus Fabricius (Coleoptera: Bruchidae). *Journal of the Entomological Research Society*, *10*(1), 27-35.

1. Recast the last sentence. It appears somehow verbose. I am suggesting something like this:

‘Our results suggest that an augmentative biological control program with the release of *A. biannulipes* after the introduction of *K. senegalensis *leaf powder is practicable for the management of *D. porcellus *in yam chips.’

Is the work clearly and accurately presented and does it cite the current literature?

Partly

If applicable, is the statistical analysis and its interpretation appropriate?

Yes

Are all the source data underlying the results available to ensure full reproducibility?

Yes

Is the study design appropriate and is the work technically sound?

Partly

Are the conclusions drawn adequately supported by the results?

Yes

Are sufficient details of methods and analysis provided to allow replication by others?

Partly

Reviewer Expertise:

NA

I confirm that I have read this submission and believe that I have an appropriate level of expertise to confirm that it is of an acceptable scientific standard, however I have significant reservations, as outlined above.

# Response

**Published:** 2024-12-16

**Authors:** YEYINOU LAURA ESTELLE LOKO

**Affiliations:** ENSBBA, UNSTIM, Dassa-Zoumè, Collines, Benin

**Many thanks for your contribution to the scientific quality of the reviewed manuscript.**

**Abstract:**

**Remark**: Change ‘leaf powder of these ….’ to ‘leaf powders of three medicinal plants’ **Response**: This has been corrected.

**Remark**: Your methodology includes two bioassays (laboratory and field conditions). Incidentally, what you have in the Abstract does not succinctly capture that. Specifically, I suggest that you should make some clarifications: “Yam chips mixed with leaf powder from each species served as negative control” What would yam chips without any treatment be? **Response**: This has been corrected.

**Remark**: The results section also fails to briefly differentiate between the laboratory and field experiments.

**Response**: This has been corrected.

**Remark**: Consider the last sentence of the Conclusion and recast to make it more comprehensive.

**Response**: This has been corrected.

**Introduction:**

**Remark**: *The last three sentences of the Introduction*

 1.Remove ‘while’ at the beginning of the sentence.

**Response**: This has been done

 2. Recast the sentence beginning with ‘Indeed, compared…’ you may wish to consider the following: Combining biological control agent with botanical insecticides is more compatible than combining it with synthetic insecticides.

**Response**: Thank you for the suggestion. This has been done Preparation of botanical powder

**Methods:**

**Remark**: To what moisture content did you dry the botanicals or ‘just until crisp’? That information will support reproducibility of the research.

**Response**: just until crisp

**Remarks**: Bioassay under laboratory conditions

 1. insert the word ‘to’. Let it read: … was assessed according to the …

**Response**: This has been corrected.

 2. Let ‘plastic boxes’ be changed to … ‘plastic experimental boxes…’ and expunge ‘experimental boxes’ in parenthesis at the end of the sentence.

**Response**: This has been corrected.

 3. Do not begin a sentence with an abbreviation. *D. porcellus* should be written in full at the beginning of a sentence to read ‘ *Dinoderus porcellus*’.

**Response**: This has been corrected.

**Remark**: Bioassays under farm storage with variable environmental conditions What of the negative control with plant powders which you mentioned in the Abstract?

**Response**: This was a mistake and has been corrected.

**Remark**: You used woven polypropylene bags for the field experiment and you claimed that the bags were hermetically sealed. How feasible is it to achieve a hermetic condition with a woven bag? Or did you put an airtight inner layer in the woven bag?

**Response**: This has been corrected

**Remark**: Did you consider the immatures in the chips?

**Response**: Yes, those that we were able to observe were counted. Data analysis.

**Remark**: In the last sentence under Data analysis, ‘The original data obtained were presented in the tables and figures’: Which data are you referring to? Is this sentence really necessary? **Response**: The sentence was removed.

**Results:**

**Remark**: Tidy up some typographical errors, omission of words; and reduce verbosity. For instance, you may wish to consider the first sentence to be recast as follows: ‘Table 1 presents percentage corrected mortalities of *D. porcellus* ….’

**Response**: This has been corrected

**Remark**: Use ‘significantly (df = 27, …..) different’. Do not omit the word ‘different’

**Response**: This has been corrected

**Remark**: Which control are you refereeing to in Table 2? Indicate it as a footnote since you have different controls for your bioassays.

**Response**: This has been mentioned.

**Remark**: The df should be 27. You typed 2 somewhere on page 4 of 12 towards the end of the second column.

**Response:** df = 27 for treatments, df = 2 for the different combinations of botanical powders and *A. biannulipes* (3 combinations)

**Remark** : Where p=0.000, I suggest that you present it as p<0.0001.

**Response**: This has been corrected

**Remark**: Be consistent with the number of decimal places. For instance, 80±9.12% should be rendered as 80.00±9.12%.

**Response**: This has been corrected

**Remark**: Does *D. porcellus* have both larvae and nymphs? Is it a holometabolous or hemimetabolous insect?

**Response **: Holometabolous insect

**Remark**: Delete the period/point after the table title or figure legend, if such is not a sentence. **Response**: This has been done.

**Discussion:**

**Remark**: First paragraph 6th line from the end of the paragraph: synthetic insecticide could be act by ..., Delete ‘be’

**Response**: This has been done.

**Remark**: Third paragraph: Use ‘spiracles’ instead of ‘stigmata’. Stigmata is more appropriate for acarina than insects.

**Response**: This has been corrected. 

**Remark**: Third paragraph: The last sentence is too clumsy. Attempt to recast it. You may wish to consider this suggestion: Therefore, it is important to determine the mechanism of lethal action of the tested botanical powders.

**Response**: Thank you for the suggestion. This has been corrected.

**Remark**: Paragraph four: Recast: … interaction did not effect on the development … Recast. **Response**: Thank you for the suggestion. This has been corrected.

**Remark**: Some studies have demonstrated the egg-laying deterrent properties of *K. senegalensis* products on the Bostrichid, *Callosobruchus maculatus Fabricius* (Bamaiyi et al. 2006) Callosobruchus maculatus is not a Bostrichid, but a Bruchid.

**Response**: This has been corrected.

**Remark**: For ‘some studies’, you need more than just one reference. You may wish to add Babarinde and Ewete (2008) to Bamaiyie et al., 2006; with the following citation: Babarinde, S. A., & Ewete, F. K. (2008). Comparative bioactivity of three Khaya species (Meliaceae) against Callosobruchus maculatus Fabricius (Coleoptera: Bruchidae). Journal of the Entomological Research Society, 10(1), 27-35.

**Response**: The reference has been added.

**Remark**: Recast the last sentence. It appears somehow verbose. I am suggesting something like this: ‘Our results suggest that an augmentative biological control program with the release of *A. biannulipes* after the introduction of K. senegalensis leaf powder is practicable for the management of D. porcellus in yam chips.’

**Response**: Thank you for the suggestion. This has been corrected.

# Reviewer report: Reviewer response for version 1

**DOI:** 10.21956/openresafrica.16338.r31525

**Published:** 2024-08-29

**Authors:** Guru P. N

**Affiliations:** 1 Department of Agricultural Entomology, Indian Council of Agricultural Research (ICAR)-Central Institute of Post-Harvest Engineering and Technology, Ludhiana, Punjab, India

Following are my comments on the manuscript...

 - The title needs to be revised. Typo corrections and clarity is needed.

 - Abstract- Background - instead of few months, mention the range of period

 - Abstract- Background - Line 4, mention the botanicals before name

 - Recheck the key words and add the suitable ones

 Under heading Mass rearing of D. porcellus- coordinates of the locations would be useful. Line 9 - instead of twenty couples write as 'twenty pairs'

 - Heading -Preparation of botanical powders, mention ambient lab conditions

 - English writing check is required. There are grammatical, typo and syntax errors. Grammatical and syntax errors may be corrected

 - Fig 1. Differentiation between B. sapida and K. senegalensis is still needed in marking lines. The lines are not clear

 - Fig 2,3,4- they have presented well. However my suggestion to the authors is that, the graphs may be revised showing the significant difference and also the comparative tukey's tests. Not a mandatory correction

 - The study is good and is of interest to readers. However, I wish to seek response from the authors regarding following points,

 1. What about any residues of these plant powders, any observations done

 2. Are there any effects of botanical powders on predator

 3. What about the sensory quality of the treated chips i.e., consumer acceptance

 4. Why only these botanicals were chosen

Is the work clearly and accurately presented and does it cite the current literature?

Partly

If applicable, is the statistical analysis and its interpretation appropriate?

Partly

Are all the source data underlying the results available to ensure full reproducibility?

Partly

Is the study design appropriate and is the work technically sound?

Yes

Are the conclusions drawn adequately supported by the results?

Partly

Are sufficient details of methods and analysis provided to allow replication by others?

Partly

Reviewer Expertise:

Agricultural Entomology

I confirm that I have read this submission and believe that I have an appropriate level of expertise to confirm that it is of an acceptable scientific standard, however I have significant reservations, as outlined above.

# Response

**Published:** 2024-12-16

**Authors:** YEYINOU LAURA ESTELLE LOKO

**Affiliations:** ENSBBA, UNSTIM, Dassa-Zoumè, Collines, Benin

**Many thanks for your contribution to the scientific quality of the reviewed manuscript.**

**Remark**:  The title needs to be revised. Typo corrections and clarity is needed.

**Response**: The title has been revised. However, it should be noted that the first letter of each insect’s genus name, order and family was written in lower case to fit the style of the journal.

**Remark**:  Abstract- Background - instead of few months, mention the range of period **Response**: This has been done.

**Remark:**  Abstract- Background - Line 4, mention the botanicals before name

**Response**: Botanicals were mentioned before name.

**Remark:** Recheck the key words and add the suitable ones

**Response**: This has been done.

**Remark:** Under heading Mass rearing of D. porcellus- coordinates of the locations would be useful.

**Response**: The coordinates of  Dantokpa market have been added.

**Remark:** Line 9 - instead of twenty couples write as 'twenty pairs'

**Response**: This has been corrected.

**Remark:** Heading -Preparation of botanical powders, mention ambient lab conditions **Response**: This has been done.

**Remark:** English writing check is required. There are grammatical, typo and syntax errors. Grammatical and syntax errors may be corrected

**Response**: This has been corrected.

**Remark:** Fig 1. Differentiation between B. sapida and K. senegalensis is still needed in marking lines. The lines are not clear

**Response**: The figure has been corrected.

**Remark:** Fig 2,3,4- they have presented well. However my suggestion to the authors is that, the graphs may be revised showing the significant difference and also the comparative tukey's tests. Not a mandatory correction

**Response**: Thank you for this suggestion.

**Remark:** The study is good and is of interest to readers. However, I wish to seek response from the authors regarding following points,

 1.What about any residues of these plant powders, any observations done

**Response**: Thank you for this remark. We considered this in the discussion and as a recommendation in the conclusion.

 2.Are there any effects of botanical powders on predator

**Response**: Line 201-206. No significant difference in terms of the survival of *A. biannulipes* exposed to botanical powders was observed compared to the positive control.

 3.What about the sensory quality of the treated chips i.e., consumer acceptance

**Response**: We recommended that sensory studies be carried out subsequently to verify whether the use of these powders has no impact on the sensory quality of the treated yam chips. 

 4.Why only these botanicals were chosen

**Response: **In the Republic of Benin, the leaves of *Bridelia ferruginea*, *Blighia sapida*, and *Khaya senegalensis* were recorded as being used as insecticides to protect yam chips (Loko et al., 2013). Line 67-69 : Furthermore, powders from the leaves of *Bridelia ferruginea* (Phyllanthaceae), *Blighia sapida* (Sapindaceae), and *Khaya senegalensis* (Meliaceae) used by farmers to control insects in stored yam chips are known to have insect repellent and insecticide properties against *D. porcellus* (Loko et al., 2017).

**Loko Y.L.**, Dansi A., Tamo M., Bokonon-Ganta A.H., Assogba P., Dansi M., Vodouhè R., Akoegninou A., Sanni A. (2013). Storage insects on yam chips and their traditional management in Northern Benin. The Scientific World Journal, vol. 2013, Article ID 484536, 11 p. http://dx.doi.org/10.1155/2013/484536.

**Loko Y.L.**, Alagbe O., Dannon EA., Datinon B., Orobiyi A., Thomas-Odjo A., Dansi A., Tamò M. (2017). Repellent effect and insecticidal activities of *Bridelia ferruginea*, *Blighia* *sapida*, and *Khaya senegalensis *leaves powders and extracts against *Dinoderus porcellus* in infested dried yam chips. Psyche: A Journal of Entomology. Article ID 5468202, 18 p. https://doi.org/10.1155/2017/5468202.
